# Supplementary material for: WDR2 regulates the orphan kinesin KIN-G to promote hook complex and Golgi biogenesis in Trypanosoma brucei
Source: mBio. 2025 May 30;16(7):e00371-25. doi: 10.1128/mbio.00371-25 (PMC12239596; doi:10.1128/mbio.00371-25)
Supplement: Figure S1 — Co-IP of WDR2 and KIN-G and predicted structures of the WDR2-KIN-G complex. [file mbio.00371-25-s0001.pdf]

Figure S1

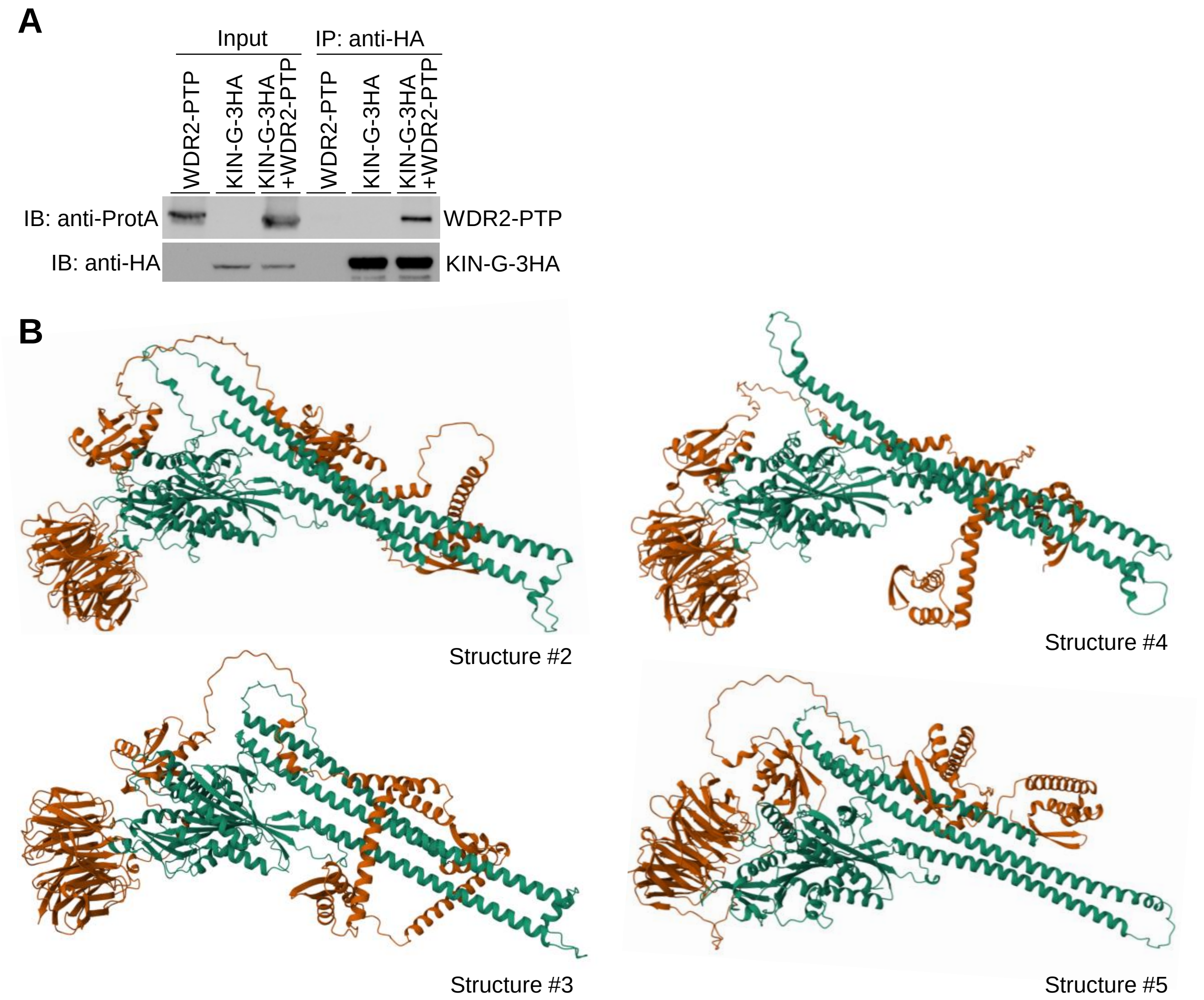

Figure S1. *In vivo* interaction between WDR2 and KIN-G and AlphaFold3-predicted structures of the WDR2-KIN-G complex. (**A**). Anti-HA immunoprecipitation to detect the *in vivo* interaction between PTP-tagged WDR2 and 3HA-tagged KIN-G in trypanosomes. IP: immunoprecipitation; IB: immunoblotting. (**B**). AlphaFold3-predicted structures of the WDR2-KIN-G complex. Shown are the other four different structures predicted by AlphaFold3.
