# Supplementary material for: WDR2 regulates the orphan kinesin KIN-G to promote hook complex and Golgi biogenesis in Trypanosoma brucei
Source: mBio. 2025 May 30;16(7):e00371-25. doi: 10.1128/mbio.00371-25 (PMC12239596; doi:10.1128/mbio.00371-25)
Supplement: Figure S2 — Mass spectrometric identification of KIN-G pulled down by WDR2. [file mbio.00371-25-s0002.pdf]

Figure S2

MAKPLSGKAAPKNISVFLRVRPPVPRELKGG**T**FNNLVCDPSDPQRVTITRGGSARKGTKSFLFNRVFDPEC**T**QQTIYNEVARGAVDAAFDGQHGVLFVYG
QTGSGKTFTISNNDPEKPGVLQQSLRDIWDRFQADTEYDYSCTVSYVQLYNEMLTDLLDPQGGRVRIQLGPEGRGDVVLVTEASGASIERKVE**S**YEDCLK
YFYEGMDRKEMTSTKMNNTSSRSHTVFNFNLTRSAKVKTVDLSSAKANNEPVIALQGRLVVCDLAGSERASRTNAEGKTLDEATHINGSLLVLGKVVAAL
TESGSQHAPFRESKLTRILQYSLLGNGNTSIVVNCSPCDDSTEETLGAIMFGQRAIQIKQDAKRHEILDYKALYYQLLADLDSKNDR**T**LETALSEERTAY
EDRIRVLEERIKIL**T**SENDMLRRESSQLGGTGPVSGTSTASGAAAAAVAMGGDDANDWRSLTMKMRRAIEKLDADLKRTDKERVELAQFLALEKNKVNVL
AQKLRAESLKHIMENKELTQRV**T**ELSIDNAKLKGTDYISFQPSAACEDALPLSLDSPRRGTPSSGLSQSINVGDAYLQEQLDKANRQLRVLNEERVELIV
YQMMASKAIRLLHAEK**T**SLANHLEKLKA

Figure S2. Mass spectrometric identification of the native KIN-G protein co-immunoprecipitated by ectopically expressed, 3HA-tagged WDR2. Peptides identified are highlighted in red. Phosphosites are underlined and listed below: Thr-32; Thr-72; Ser-194; Thr-301; Thr-388; Thr-415; Thr-523; Thr-617.
